# Preexisting Diabetes and Breast Cancer Treatment Among Low-Income Women

**DOI:** 10.1001/jamanetworkopen.2024.9548

**Published:** 2024-05-08

**Authors:** Bayu Begashaw Bekele, Min Lian, Chester Schmaltz, Tracy Greever-Rice, Pratibha Shrestha, Ying Liu

**Affiliations:** 1 Division of Public Health Sciences, Department of Surgery, Washington University School of Medicine, St Louis, Missouri; 2 Alvin J. Siteman Cancer Center, Washington University School of Medicine, St Louis, Missouri; 3 Division of General Medical Sciences, Department of Medicine, Washington University School of Medicine, St Louis, Missouri; 4 Department of Health Management and Informatics, University of Missouri School of Medicine, Columbia; 5 Center for Health Policy, University of Missouri, Columbia

## Abstract

**IMPORTANCE:**

Diabetes is associated with poorer prognosis of patients with breast cancer. The association between diabetes and adjuvant therapies for breast cancer remains uncertain.

**OBJECTIVE:**

To comprehensively examine the associations of preexisting diabetes with radiotherapy, chemotherapy, and endocrine therapy in low-income women with breast cancer.

**DESIGN, SETTING, AND PARTICIPANTS:**

This population-based cohort study included women younger than 65 years diagnosed with nonmetastatic breast cancer from 2007 through 2015, followed up through 2016, continuously enrolled in Medicaid, and identified from the linked Missouri Cancer Registry and Medicaid claims data set. Data were analyzed from January 2022 to October 2023.

**EXPOSURE:**

Preexisting diabetes.

**MAIN OUTCOMES AND MEASURES:**

Logistic regression was used to estimate odds ratios (ORs) of utilization (yes/no), timely initiation (≤90 days postsurgery), and completion of radiotherapy and chemotherapy, as well as adherence (medication possession ratio ≥80%) and persistence (<90–consecutive day gap) of endocrine therapy in the first year of treatment for women with diabetes compared with women without diabetes. Analyses were adjusted for sociodemographic and tumor factors.

**RESULTS:**

Among 3704 women undergoing definitive surgery, the mean (SD) age was 51.4 (8.6) years, 1038 (28.1%) were non-Hispanic Black, 2598 (70.1%) were non-Hispanic White, 765 (20.7%) had a diabetes history, 2369 (64.0%) received radiotherapy, 2237 (60.4%) had chemotherapy, and 2505 (67.6%) took endocrine therapy. Compared with women without diabetes, women with diabetes were less likely to utilize radiotherapy (OR, 0.67; 95% CI, 0.53-0.86), receive chemotherapy (OR, 0.67; 95% CI, 0.48-0.93), complete chemotherapy (OR, 0.71; 95% CI, 0.50-0.99), and be adherent to endocrine therapy (OR, 0.71; 95% CI, 0.56-0.91). There were no significant associations of diabetes with utilization (OR, 0.95; 95% CI, 0.71-1.28) and persistence (OR, 1.09; 95% CI, 0.88-1.36) of endocrine therapy, timely initiation of radiotherapy (OR, 1.09; 95% CI, 0.86-1.38) and chemotherapy (OR, 1.09; 95% CI, 0.77-1.55), or completion of radiotherapy (OR, 1.25; 95% CI, 0.91-1.71).

**CONCLUSIONS AND RELEVANCE:**

In this cohort study, preexisting diabetes was associated with subpar adjuvant therapies for breast cancer among low-income women. Improving diabetes management during cancer treatment is particularly important for low-income women with breast cancer who may have been disproportionately affected by diabetes and are likely to experience disparities in cancer treatment and outcomes.

## Key Points

**Question:**

Is preexisting diabetes associated with utilization, timely initiation, adherence, persistence and continuation, and/or completion of adjuvant therapies in low-income women with breast cancer?

**Findings:**

In a population-based cohort of 3704 Medicaid-insured women (aged <65 years) with breast cancer, diabetes was associated with lower likelihoods of radiotherapy use, chemotherapy use and completion, and endocrine therapy adherence. There were no significant differences in utilization and persistence of endocrine therapy, timely initiation of radiotherapy and chemotherapy, or completion of radiotherapy between women with and without diabetes.

**Meaning:**

These findings suggest interventions optimizing diabetes management during breast cancer treatment may also improve cancer care for low-income patients.

## Teaser

This cohort study evaluates the associations of preexisting diabetes with radiotherapy, chemotherapy, and endocrine therapy in low-income women with breast cancer.

## Introduction

Breast cancer is the most common malignant neoplasm among women in the US, accounting for 31% of all types of cancers.^1^ The American Society of Clinical Oncology and National Comprehensive Cancer Network highly recommends radiotherapy, endocrine therapy (for hormone receptor-positive breast cancer), and chemotherapy after resection of breast cancer.^2,3^ Despite improvements in screening and treatment, breast cancer remains the second leading cause of cancer-related deaths in women.^1^ Moreover, advances in breast cancer treatment have not equally benefited all patients, with a longstanding socioeconomic disparity for treatment and prognosis. Low socioeconomic status is associated with higher risks of late-stage diagnosis and treatment underuse for breast cancer,^4,5,6^ which collectively contribute to poorer survival in socioeconomically disadvantaged patients.^7,8^ Medicaid is the primary program providing comprehensive health care coverage to low-income people. While Medicaid expansion under the Affordable Care Act has been associated with early detection and high-quality treatment for breast cancer,^9,10,11^ Medicaid coverage alone is insufficient for low-income patients to receive guideline-recommended breast cancer treatment and consequently improve their outcomes. Compared with their privately insured counterparts, Medicaid-insured women with breast cancer are more likely to be diagnosed at more advanced stages and have treatment delays^12^ and less likely to receive stage-appropriate treatment^13^ and complete adjuvant therapy.^14^ Less is known about the factors influencing breast cancer treatment among Medicaid beneficiaries.

Comorbid conditions play a critical role in the choice of breast cancer treatment.^15,16,17^ Diabetes is one of the top prevalent comorbidities in women with breast cancer and affects up to one-third of patients.^18,19^ The adverse impact of preexisting diabetes on breast cancer survival has been well documented and may result in less intensive cancer care.^19,20^ Studies have consistently reported a lower likelihood of receiving chemotherapy for breast cancer in women with diabetes than women without diabetes, especially among patients younger than 65 years.^20,21,22,23^ The role of diabetes in utilization of radiotherapy and endocrine therapy for breast cancer remains unclear. Among women with breast cancer identified from 7 US state cancer registries, moderate-to-severe diabetes was associated with underuse of radiotherapy following breast-conserving surgery in the group aged younger than 65 years, but not the group aged 65 years and older.^21^ By contrast, a Dutch population-based study showed a significant association between diabetes and radiotherapy underuse in older women with breast cancer, which was not seen in younger patients.^23^ Similarly, prior analyses yielded mixed results for diabetes and endocrine therapy for breast cancer. We previously found that comorbid diabetes was associated with a lower likelihood of endocrine therapy among women with hormone receptor-positive breast cancer treated at comprehensive cancer and medical centers,^24^ while a population-based study reported a higher level of endocrine therapy in patients with diabetes aged younger than 65 years and a similar level in patients with diabetes aged 65 years and older compared with their counterparts without diabetes.^23^ Additionally, prior studies assessed only 1 or 2 types of therapies, which only constituted part of a cancer treatment plan. All of these studies examined adjuvant therapies as binary variables (yes or no) with no focus on whether patients actually completed radiotherapy and chemotherapy and whether they adhered to and persisted in endocrine therapy, which are also important contributors to breast cancer prognosis.^25,26,27^

To fill these knowledge gaps, we comprehensively examined the associations of preexisting diabetes with utilization, timely initiation, adherence, persistence and continuation, and/or completion of adjuvant therapies for breast cancer among Medicaid-insured women. Understanding the factors influencing cancer treatment among Medicaid beneficiaries is critical for interventions to improve breast cancer care in this medically vulnerable and underserved population, thereby reducing socioeconomic disparities in breast cancer.

## Methods

### Data Sources

Using Medicaid claims, we identified women enrolled in the Medicaid fee-for-service program and diagnosed with breast cancer in Missouri between 2007 and 2016. For these patients, their Medicaid claims for emergency, inpatient, and outpatient services and prescribed medications were linked with Missouri Cancer Registry data using Link Plus, version 2.0 (US Centers for Disease Control and Prevention). A probabilistic matching approach was applied, and matching variables included unique departmental control numbers, first and last names, social security number, date of birth, and race and ethnicity.^28,29^ Adhering to the data collection and coding guidelines established by the National Program of Cancer Registries, the Missouri Cancer Registry regularly collects the demographic (age, race and ethnicity, sex, and census tract at diagnosis), tumor (date of diagnosis, primary site, cancer stage, tumor grade, tumor size, and hormone receptor status), and cancer-related treatment (dates of definitive surgery, first radiotherapy, first chemotherapy, and first endocrine therapy) for more than 95% of all incident cases in Missouri. This cohort study was approved by the institutional review boards of Washington University School of Medicine in St. Louis, the Missouri Department of Health and Senior Services, and the Missouri Department of Social Services with a waiver of consent due to the use of deidentified data. We followed the Strengthening the Reporting of Observational Studies in Epidemiology (STROBE) reporting guidelines.^30^

### Patient Selection

**Figure 1.**
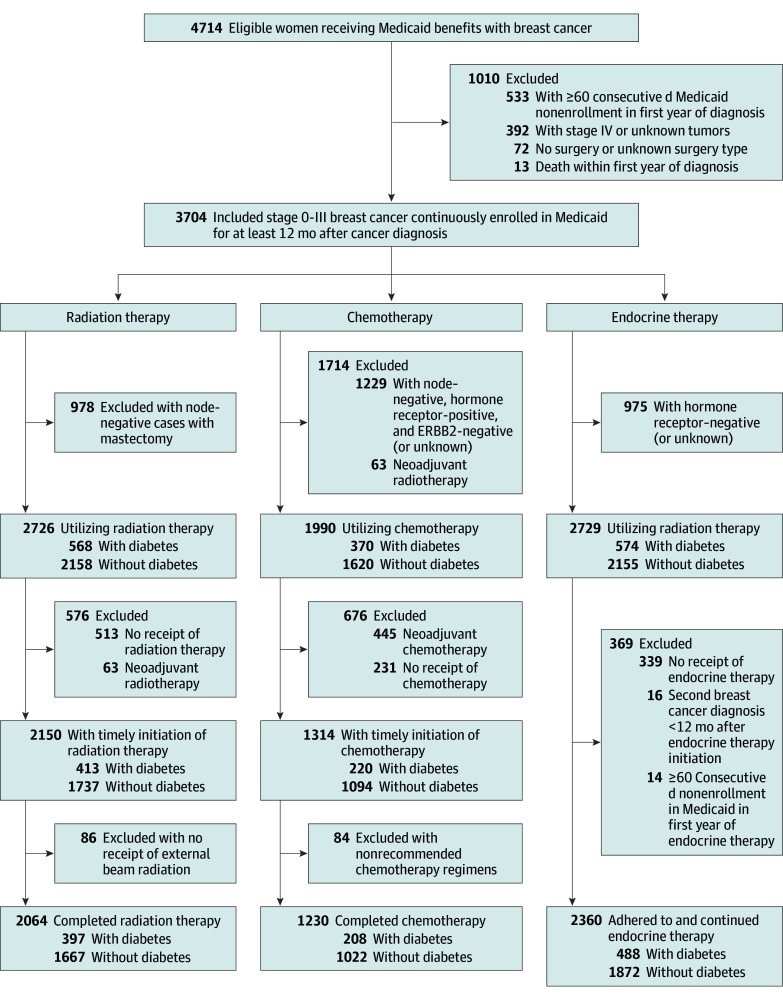
Flowchart Outlining the Selection of Study Samples for Each Analysis

The study included women enrolled in Medicaid and diagnosed with breast cancer as a first primary malignant neoplasm before age 65 years between January 2007 and December 2015. Continuous enrollment in Medicaid was defined as fewer than 60 consecutive days of nonenrollment status per enrollment year.^29^ We excluded women who were not continuously enrolled in Medicaid in the first year of diagnosis, had stage IV or unknown stage tumors, did not undergo breast-conserving surgery or mastectomy, or died in the first year of diagnosis. There were 3704 cases eligible for the study. Figure 1 shows further exclusion criteria and final sample size for each analysis.

### Diabetes and Covariates

A diagnosis of diabetes was determined based on claims from 12 months before to a month after cancer diagnosis. Diabetes-related claims were identified using *International Statistical Classification of Diseases and Related Health Problems, Ninth *and *Tenth Revision (ICD-9* and *ICD-10)* codes for primary diabetes (250.x, E10, and E11). Demographic variables included age (≤50, 50-54, 55-59, and 60-64 years) and self-reported race and ethnicity (non-Hispanic Black, non-Hispanic White, and other [American Indian, Asian, Hispanic, some other race, and missing; some other race was one of the race categories defined by the Missouri Cancer Registry, and we do not know the groups included in this category]). Due to racial and ethnic differences in breast cancer treatment, race and ethnicity were included as a covariate. Original racial and ethnic categories were defined by the Missouri Cancer Registry and are consistent with the US Census. The composite socioeconomic deprivation index for each census tract was calculated based on 21 variables from 2005 to 2009 (for cases with 2000 residential census tract code available) and 2008 to 2012 (for cases with 2010 residential census tract code available) American Community Surveys.^31,32,33^ The index scores were categorized into quartiles, with a higher quartile indicating greater socioeconomic deprivation. The rural census tracts were defined as nonmetropolitan areas and identified using the rural-urban commuting area codes from the US Department of Agriculture.^33^ Clinical covariates included cancer stage (0, I, II, and III), breast cancer subtypes (hormone receptor–positive, hormone receptor–negative, and missing), and type of definitive surgery (breast-conserving surgery and mastectomy).

### Adjuvant Therapies

We identified radiotherapy using the *Current Procedural Terminology*, *Healthcare Common Procedure Coding System*, and *ICD* codes for radiation treatment delivery that was claimed within a year of diagnosis (eTable 1 in Supplement 1). Time to radiotherapy was calculated as the interval between the date of definitive surgery and the date of first radiotherapy and was dichotomized as less than 90 or 90 or more days because a delay greater than 90 days was associated with increased risks of ipsilateral breast cancer recurrence and mortality.^34,35^ For patients who initiated external beam radiotherapy, the total number of radiation sessions was counted during a 90-day period from the first radiotherapy. The cutoffs of 15 sessions for radiotherapy following breast-conserving surgery and 25 sessions for radiotherapy following mastectomy were used to define radiotherapy completion.^14^

A patient was considered having received chemotherapy if there was a claim for chemotherapy delivery within a year of diagnosis (eTable 1 in Supplement 1). Time to chemotherapy was calculated similarly to time to radiotherapy. Chemotherapy delay was defined as an interval of more than 90 days because chemotherapy initiated more than 90 days post-surgery was associated with increased mortality.^25,36^ The duration of chemotherapy was measured as the number of weeks between the first and last claims for chemotherapy. eTable 2 in Supplement 1 shows the guideline-recommended chemotherapy regimens that were used for Medicaid patients. Treatment is typically administered every 2 to 4 weeks for 4 to 8 cycles.^2^ We defined a complete course of chemotherapy as completion of 3 to 7 cycles (depending on the regimen a patient received) to account for missing claims.

Initiation of endocrine therapy was defined as a single prescription of tamoxifen, toremifene, anastrozole, letrozole, or exemestane on the basis of national drug codes within a year of breast cancer diagnosis. Dates of service and days’ supply of medications were used to calculate the number of pills supplied over 12 months following the initial prescription. Adherence was measured by the medication possession ratio (≥80%) that was estimated as the percentage of total days supply of medications in a year.^29^ Persistence and continuation was defined as having no gap in medication supply for at least 90 days in the first year of treatment.^29^

### Statistical Analysis

Logistic regression was applied to estimate odds ratios (ORs) of utilization, timely initiation, adherence, persistence and continuation, and/or completion of adjuvant therapies. Models were adjusted for age, race and ethnicity, neighborhood socioeconomic deprivation, urban-rural location, cancer stage, and type of surgery. Statistical analyses were conducted using SAS version 9.4 (SAS Institute). A 2-sided *P*-value less than .05 was considered statistically significant. Analyses were performed in January 2022 to October 2023.

## Results

Among 3704 patients, the mean (SD) age was 51.4 (8.6) years, 1038 (28.1%) were non-Hispanic Black, 2598 (70.1%) were non-Hispanic White, 765 (20.7%) had preexisting diabetes, 2369 (64.0%) received radiotherapy, 2237 (60.4%) had chemotherapy, and 2505 (67.6%) took endocrine therapy. Compared with women without diabetes, women with diabetes were older and were more likely to be non-Hispanic Black, live in deprived neighborhoods, have pathologically favorable tumors, and undergo breast-conserving surgery (Table). There was no difference in rural locations between the 2 groups.

**Table.** Characteristics of Medicaid-Insured Women With Breast Cancer in Missouri by Diabetes Status

Characteristics	Patients, No. (%)	
	With diabetes (n = 765)	Without diabetes (n = 2939)
Age, y		
≤50	146 (19.1)	1279 (43.5)
50-54	167 (21.8)	601 (20.5)
55-59	201 (26.0)	572 (19.5)
60-64	251 (34.0)	487 (16.6)
Race		
Non-Hispanic Black	260 (34.0)	778 (26.5)
Non-Hispanic White	493 (64.4)	2105 (71.6)
Other	10 (1.3)^a^	46 (1.6)^b^
Missing	2 (0.3)	10 (0.3)
Census tract–level socioeconomic deprivation, quartile		
1 (Least)	75 (9.8)	373 (12.7)
2	179 (23.4)	632 (21.5)
3	203 (26.5)	851 (29.0)
4 (Most)	296 (38.7)	1023 (34.8)
Missing	12 (1.6)	60 (2.0)
Rural residence		
Urban	553 (72.3)	2120 (72.1)
Rural	197 (25.8)	743 (25.3)
Missing	15 (2.0)	76 (2.6)
Cancer stage		
0	119 (15.6)	366 (12.5)
I	262 (34.3)	839 (28.6)
II	289 (37.8)	1184 (40.3)
III	95 (12.4)	550 (18.7)
Tumor grade		
Well-differentiated	137 (17.9)	465 (15.8)
Moderately differentiated	295 (38.6)	1065 (36.2)
Poorly differentiated or undifferentiated	291 (38.0)	1271 (43.3)
Missing	42 (5.5)	138 (4.7)
Hormone receptor status		
Positive	574 (75.0)	2155 (73.3)
Negative	158 (20.7)	678 (23.1)
Undetermined	33 (4.3)	106 (3.6)
Type of definitive surgery		
Breast conserving surgery	393 (51.4)	1313 (44.7)
Mastectomy	372 (48.6)	1626 (55.3)

^a^ Included an American Indian patient, 7 Asian patients, and 2 Hispanic patients.

^b^ Included 2 American Indian patients, 28 Asian patients, 5 Hispanic patients, 3 White patients with no information about Hispanic origin, and 8 patients of some other race. Some other race was one of the race categories defined by the Missouri Cancer Registry, and the groups included in this category are not known.

**Figure 2.**
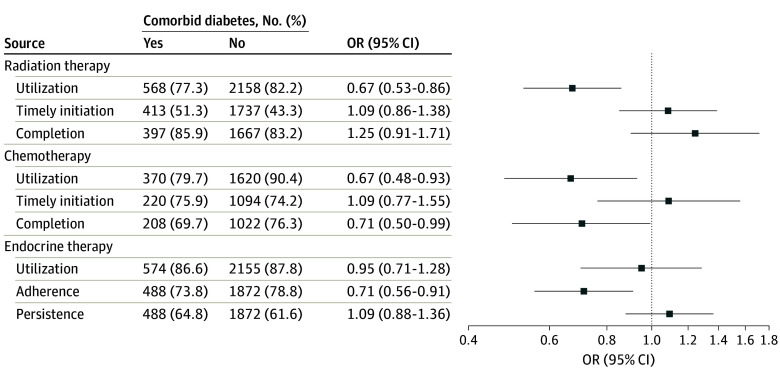
Associations of Diabetes With Adjuvant Therapies for Breast Cancer Among Medicaid-Insured Women The analysis of radiation therapy was restricted to patients undergoing breast-conserving surgery and node-positive patients undergoing mastectomy. Chemotherapy was analyzed in patients with node-positive tumors and patients with hormone receptor-negative tumors or hormone receptor-positive and ERBB2-positive tumors. The analysis of endocrine therapy was limited to patients with hormone receptor-positive tumors. The OR and 95% CI were adjusted for age, race and ethnicity, census tract-level socioeconomic deprivation, rural residency, cancer stage, and type of surgical treatment. OR indicates odds ratio.

Figure 2 shows utilization, timely initiation, adherence, persistence and continuation, and/or completion of adjuvant therapies for breast cancer in patients with diabetes and patients without diabetes. Among cases undergoing breast-conserving surgery and node-negative cases undergoing mastectomy (2726 participants), 439 of 568 of women with diabetes (77.3%) and 1774 of 2158 of women without diabetes (82.2%) received radiotherapy, respectively. Radiotherapy was initiated within 90 days post-surgery in 212 of 413 patients with diabetes (51.3%) and 752 of 1737 patients without diabetes (43.3%). Among 2064 patients with external beam radiotherapy, the majority (314 of 397 patients with diabetes [85.9%] and 1387 of 1667 patients without diabetes [83.2%]) completed the treatment. Compared with patients without diabetes, patients with diabetes had a significantly lower likelihood of radiotherapy (OR, 0.67; 95% CI, 0.53-0.86) after adjustment for covariates. Diabetes was not significantly associated with timely initiation (OR, 1.09; 95% CI, 0.86-1.38) or completion (OR, 1.25; 95% CI, 0.91-1.71) of radiotherapy.

Among 1990 patients with node-positive, hormone receptor-negative, or ERBB2-positive invasive breast cancer, 295 of 370 women (79.7%) with diabetes and 1464 of 1620 women (90.4%) without diabetes received chemotherapy. Moreover, 167 of 220 women (75.9%) with diabetes and 812 of 1094 women (74.2%) without diabetes started chemotherapy within 90 days after surgery. Guideline-recommended postsurgical chemotherapy regimens were identified in 1230 cases, of which 145 of 208 women (69.7%) with diabetes and 780 of 1022 women (76.3%) without diabetes completed the treatment. Diabetes was associated with significantly lower odds of utilization (OR, 0.67; 95% CI, 0.48-0.93) and completion (OR, 0.71; 95% CI, 0.50-0.99) of chemotherapy after adjustment for covariates, although timely initiation of treatment was similar between the 2 groups (OR, 1.09; 95% CI, 0.77-1.55).

The majority of patients with diabetes (497 of 574 patients [86.6%]) and patients without diabetes (1893 of 2155 patients [87.8%]) with hormone receptor-positive breast cancer (2729 patients) initiated endocrine therapy within a year of diagnosis. Overall, 360 of 488 women (73.8%) with diabetes and 1475 of 1872 women (78.8%) without diabetes were adherent in the first year after therapy initiation, and 316 of 488 women (64.8%) with diabetes and 1153 of 1872 women (61.6%) without diabetes were persistent to therapy. After adjustment for covariates, the odds of adherence were significantly lower in women with diabetes than women without diabetes (OR, 0.71; 95% CI, 0.56-0.91). There was no significant difference in endocrine therapy initiation (OR, 0.95; 95% CI, 0.71-1.28) or persistence and continuation (OR. 1.09; 95% CI, 0.88-1.36) between women with diabetes and women without diabetes.

## Discussion

Given that diabetes disproportionately affects low-income adults^37^ and also increases the risk of breast cancer,^38^ understanding the role of diabetes in adjuvant therapy for breast cancer among low-income women is critical to inform the appropriate care of this medically vulnerable patient population. In this population-based study, we comprehensively evaluated utilization, timely initiation, adherence, persistence and continuation, and/or completion of adjuvant therapies for breast cancer among Medicaid-insured women aged less than 65 years with and without diabetes. We found significant associations between preexisting diabetes and adjuvant therapies. Compared with women without diabetes, women with diabetes were less likely to receive radiotherapy and chemotherapy. Among patients who initiated chemotherapy, preexisting diabetes was not associated with timeliness of treatment initiation but was associated with higher risk of treatment incompletion. While use and persistence of endocrine therapy were similar between patients with diabetes and patients without diabetes, patients with diabetes had a higher risk of nonadherence to endocrine therapy in the first year of treatment.

Underuse of chemotherapy has been reported for women with diabetes with breast cancer compared with women without diabetes with breast cancer,^20,21,22^ especially among younger patients.^23^ This may be related to higher perceived risk of chemotherapy complications in patients with diabetes. Importantly, we extended the literature by examining the association of diabetes with timely initiation and completion of chemotherapy. Our finding of no association between diabetes and timeliness of chemotherapy initiation suggests that if patients decided to take chemotherapy, the treatment was delivered within 90 days post-surgery for most of them, regardless of their diabetes history. However, our study also revealed that patients with diabetes receiving chemotherapy were less likely to have a complete course of treatment compared with patients without diabetes. This finding is in line with studies showing that diabetes increased the risks of hospitalization and emergency department visits for chemotherapy toxicity (eg, infection, neutropenia, and anemia).^22,39^

Consistent with other studies,^20,21^ our study among Medicaid patients aged less than 65 years showed that diabetes was associated with underuse of radiotherapy for breast cancer. In contrast to our study, van de Poll-Franse et al^23^ observed a lower use of radiotherapy in older, but not younger, Dutch patients with breast cancer and diabetes compared with their counterparts without diabetes. The authors noted that lower odds of undergoing breast-conserving surgery in older patients with diabetes (vs without diabetes) in the study might explain a lower use of adjuvant radiotherapy. In a population-based study in New Zealand, women with diabetes with breast cancer had lower odds of radiotherapy following lumpectomy compared with counterparts without diabetes.^40^ This difference was not significant after adjustment for comorbidity scores in addition to demographic and tumor factors. Considering high correlations between diabetes and other comorbidities, a null finding of that study might result from over-adjustment for comorbidity scores. Among patients who received radiotherapy, we did not find any significant difference in timeliness of radiotherapy initiation or treatment completion between women with and without diabetes. This suggests that patients with diabetes who initiated radiotherapy might have knowledge of managing potential adverse effects of radiotherapy.

Endocrine therapy use, adherence, and persistence are a particularly critical issue in breast cancer care because almost 80% of breast cancers are hormone receptor-positive^41^ and could benefit from endocrine therapy. The current study aligns with other population-based studies, showing no significant difference in endocrine therapy use between women with and without diabetes.^21,42^ Conversely, a Dutch population-based study revealed that endocrine therapy use was higher among patients with breast cancer younger than 65 years with diabetes than those without diabetes.^23^ However, that study did not take hormone receptor status into consideration, and diabetes has been associated with a higher incidence of hormone receptor-positive breast cancer.^43^ Among patients with breast cancer treated at a comprehensive cancer center and another academic medical center, we previously observed a lower use of endocrine therapy in hormone receptor-positive patients with diabetes vs without diabetes.^24^ The discrepancy in findings might be partially attributable to differences in study design and patient characteristics; for example, patients who reported Black race accounted for nearly 41% in that study and only 6% to 28% in the current and other studies. A higher burden of diabetes in Black people^37^ and a lower use of endocrine therapy in Black women with breast cancer^44^ might collectively contribute to a finding of diabetes-associated higher risk of endocrine therapy underuse in that study.

Among the patients who initiated endocrine therapy in the current study, approximately a quarter did not take medications more than 20% of days and more than 35% had at least 90 days without prescription refills in the first year of treatment, which are comparable with or lower than the nonadherence and nonpersistence rates reported for Medicaid-insured women with breast cancer.^45,46,47^ Of note, we found an inverse association between preexisting diabetes and endocrine therapy adherence in the first year, which is particularly relevant to interventions addressing an issue of low adherence during the 5-year treatment because of a critical role of adherence in the first year in maintaining adherence for subsequent years.^29^ A meta-analysis showed that the risk of type 2 diabetes was increased by 30% in patients with breast cancer who received endocrine therapy compared with patients with breast cancer who did not use endocrine therapy and by 19% compared with matched cancer-free control individuals.^48^ Therefore, endocrine therapy could make the control of glucose levels more challenging for patients with breast cancer and preexisting diabetes, which might explain their higher risk of nonadherence to endocrine therapy.

### Limitations

This study has limitations. First, it was restricted to patients aged younger than 65 years enrolled in Medicaid. Therefore, the results may not be generalizable to older patients and patients with other types of health insurance. Second, we evaluated adherence and persistence and continuation of endocrine therapy in the first year of treatment, which may not reflect the long-term association between diabetes and endocrine therapy. Third, we defined completion of adjuvant radiotherapy and chemotherapy solely based on administrative claims. Additionally, we could not account for physician characteristics that might contribute to their patients’ treatment choice and completion.

## Conclusions

This cohort study suggests the inverse associations of preexisting diabetes with initiation, adherence, and/or completion of adjuvant radiotherapy, chemotherapy, and endocrine therapy among low-income women with breast cancer. Future research should identify the optimal management of diabetes during breast cancer treatment. Integration of nurse practitioner–led management of diabetes into multidisciplinary breast cancer care approaches has been proposed to address special medical needs of patients with both breast cancer and diabetes.^49^ This is particularly important for low-income patients with breast cancer, as they have a higher prevalence of diabetes, suboptimal cancer treatment, and poorer prognosis.

## Notes

Supplement 1. **eTable 1. **Codes in the Medicaid Claims for Radiotherapy and Chemotherapy **eTable 2. **Guideline-Recommended Chemotherapy Regimens Used in Medicaid Patients With Breast Cancer

Supplement 2.
Data Sharing Statement
