# Supplementary material for: Preexisting Diabetes and Breast Cancer Treatment Among Low-Income Women
Source: JAMA Netw Open. 2024 May 8;7(5):e249548. doi: 10.1001/jamanetworkopen.2024.9548 (PMC11079686; doi:10.1001/jamanetworkopen.2024.9548)
Supplement: Supplement 1. — eTable 1. Codes in the Medicaid Claims for Radiotherapy and Chemotherapy eTable 2. Guideline-Recommended Chemotherapy Regimens Used in Medicaid Patients With Breast Cancer [file jamanetwopen-e249548-s001.pdf]

Supplemental Online Content

Bekele BB, Lian M, Schmaltz C, Greever-Rice T, Shrestha P, Liu Y. Preexisting diabetes and breast cancer treatment among low-income women. *JAMA Netw Open*. 2024;7(5):e249548. doi:10.1001/jamanetworkopen.2024.9548

eTable 1. Codes in the Medicaid Claims for Radiotherapy and Chemotherapy

eTable 2. Guideline-Recommended Chemotherapy Regimens Used in Medicaid Patients With Breast Cancer

This supplemental material has been provided by the authors to give readers additional information about their work.

© 2024 Bekele BB et al. *JAMA Network Open.*

eTable 1. Codes in the Medicaid Claims for Radiotherapy and Chemotherapy

	CPT codes	HCPCS codes	ICD codes	Notes
Radiation therapy	77371-77373, 77385, 77386, 77401-77404, 77406-77409, 77411-77414, 77416-77418, 77424, 77425, 77520, 77522, 77523, 77525, 77750, 77761-77763, 77767, 77768, 77770-77772, 77776-77778, 77781-77787, 77789, 77799, 0394T, 0395T.	G6003-G6014, G6015, G6016, G0173, G0251, G0339, G0340.	V58.0, V66.1, V67.1, Z51.0, Z51.89, Z08, 92.21-92.29	CPT/HCPCS codes for external beam radiotherapy: 77402-77404, 77406-77409, 77411-77414, 77416, G6003-G6014
Chemotherapy	96400-96549	Doxorubicin: C9415, J9000-J9002, Q2048-Q2050<br>Cyclophosphamide: C9420, C9421, J8530, J9070, J9080, J9090-J9097<br>Paclitaxel: C9431, J9264, J9265, J9267, C9127<br>Docetaxel: J9170, J9171<br>Capecitabine: J8520, J8521<br>Methotrexate: J8610, J9250, J9260<br>Fluorouracil: J9190<br>Epirubicin: J9178, J9180<br>Carboplatin: J9045	V58.1, Z51.11, 99.25,	National drug codes for chemotherapy

eTable 2. Guideline-Recommended Chemotherapy Regimens Used in Medicaid Patients With Breast Cancer

Regimens	Description
TC	Docetaxel and cyclophosphamide, 4 21-day cycles
AC-T	Doxorubicin and cyclophosphamide, 4 14-day cycles, followed by paclitaxel, 4 14-day cycles
FEC	Cyclophosphamide, epirubicin, and fluorouracil, 3 21-day cycles
TCH	Docetaxel and carboplatin, 6 21-day cycles
AC	Doxorubicin and cyclophosphamide, 4 14-day cycles
TAC	Docetaxel, doxorubicin, and cyclophosphamide, 6 21-day cycles
FAC	Fluorouracil, doxorubicin, and cyclophosphamide, 6 21-day cycles
CMF	Cyclophosphamide, methotrexate, and fluorouracil, 6 28-day cycles
EC	Epirubicin and cyclophosphamide, 8 21-day cycles

© 2024 Bekele BB et al. *JAMA Network Open.*
