# Supplementary material for: Preexisting Diabetes and Breast Cancer Treatment Among Low-Income Women
Source: JAMA Netw Open. 2024 May 8;7(5):e249548. doi: 10.1001/jamanetworkopen.2024.9548 (PMC11079686; doi:10.1001/jamanetworkopen.2024.9548)
Supplement: Supplement 2. — Data Sharing Statement [file jamanetwopen-e249548-s002.pdf]

Data Sharing Statement

Bekele. Preexisting Diabetes and Breast Cancer Treatment Among Low-Income Women.
JAMA Netw Open. Published May 08, 2024. doi:10.1001/jamanetworkopen.2024.9548

Data
Data available: Yes
Data types: Other (please specify)
Additional Information: The original datasets used in this study were provided by the Missouri Cancer Registry and the MO HealthNet (Medicaid) Program with permission. Data will be shared on request to the corresponding author with permission of the Missouri Cancer Registry and the MO HealthNet.
How to access data: yliu3@wustl.edu
When available: With publication

Supporting Documents
Document types: None

Additional Information
Who can access the data: Researchers whose proposed use of the data has been approved
Types of analyses: For a specified purpose
Mechanisms of data availability: After approval of a proposal by the corresponding author of this study, the Missouri Cancer Registry, and the MO HealthNet.
